# Hydro-ecological controls on riverine organic carbon dynamics in the tropical monsoon region

**DOI:** 10.1038/s41598-019-48208-y

**Published:** 2019-08-15

**Authors:** Qianzhu Zhang, Zhen Tao, Zanwen Ma, Quanzhou Gao, Haojun Deng, Peng Xu, Jian Ding, Zhengang Wang, Youwen Lin

**Affiliations:** 1 Chongqing Branch Institute, Changjiang River Scientific Research Institute, Chongqing, 400026 China; 2 School of Geography and Planning, Sun Yat-Sen University, Guangdong Provincial Key Laboratory for Urbanization and Geo-simulation, Guangzhou, 510275 China; 3 Xinning Middle School, Taishan, 529200 China; 4 Key Laboratory of Mineral Resource & Geological Processes of Guangdong Province, Guangzhou, 510275 China; 5 Hydrology and Water Resources Survey Bureau of Hainan Province, Hainan, 570100 China

**Keywords:** Carbon cycle, Carbon cycle

## Abstract

Transport fluxes and properties of riverine organic carbon in the tropical monsoon region were the vital parameters in the global riverine organic carbon fluxes budget. The study focused on the riverine organic carbon in the Changhuajiang River (CHJR), locating at the mid-west of the Hainan Island, China. Dissolved organic carbon (DOC) concentrations in the CHJR ranged from 0.22 mg/L to 11.75 mg/L with an average of 1.75 mg/L, which was lower than the average of global rivers and had a significantly temporal and spatial variation. Output flux of riverine DOC was calculated as 0.55 t/km^2^/y, which could be revised up to 1.03 t/km^2^/y, considering that the riverine discharge before dam construction. A linear model of riverine DOC flux suitable in CHJR basin was established, which involved the factors, such as soil organic carbon, runoff depth and slope, etc. There was a large variation of POC concentrations in the CHJR where the average POC concentration in the dry season was 2.41 times of the wet season. Riverine POC flux in CHJR basin was calculated as 1.78 t/km^2^/y, higher than the average of global rivers and far lower than those in other domestic larger rivers. About 8.28 × 10^3^ t POC were exported yearly in CHJR, of which, 7.15 × 10^3^ t originated from terrestrial ecosystem and 1.13 × 10^3^ t stemmed from aquatic ecosystem. Meanwhile, about 87.74% of terrestrial source happened in the wet season and 12.26% in the dry season. This research revealed that the riverine organic carbon mainly stemmed from the surface erosion processes in the drainage basin during the wet season.

## Introduction

As the key channel linking marine and terrestrial ecosystem, global rivers played an important role on the global carbon cycle^1,2^. According to previous statistics, about 1Gt C were annually transported into the ocean via global rivers^3^, accounting for 20% of the total absorption amount (5.0Gt) in terrestrial and marine ecosystem (http://co2now.org). In the fluvial fluxes, the 40% were transported in the form of organic carbon by global rivers^4^, approximating to 1~2% of the net primary production^5^. While, the riverine organic carbon concentrations and their output process reflected terrestrial ecosystem changes, and the fluxes exported into the ocean were the important constraint of the oceanic carbon budget and cycling^6^. In previous research, two approaches that carbon data for large rivers and the model formulas were recognized to estimate global riverine carbon fluxes^7^. In the former, the measured data of riverine organic carbon concentrations in various regions was particularly important, considering that there were large differences in river organic carbon concentrations in different latitudes regions^1^. The available studies research certificated that the riverine dissolved organic carbon (DOC) concentrations were not high in the tropical regions due to the rapidly decomposition of organic matters, in spite of high primary productivities^1^. However, Huang *et al*. had pointed out that the total fluxes of riverine organic carbon in the Asia area kept at a much higher level^7^. It was possible that particulate organic carbon (POC), which was carried by particulate suspension, occupied a large proportion, but enough supporting materials should be provided. In the latter, the parameters applied for establishing fluvial fluxes models were sometimes regional, which hardly represented the overall situation of global rivers. As the results concluded by Ludwig *et al*., the drainage intensity, basin slope and amount of organic soil carbon were the main determinants of DOC fluxes and the total mass of suspended matter govern POC fluxes^4^. Based on above-mentioned models, the global riverine organic carbon fluxes were calculated and their 45% was derived from the tropical region (42.7% of land area on earth), where 61% of the total terrestrial primary productivities, 66.2% of riverine discharge and 73.2% of riverine total suspended solid (TSS) were produced^8,9^. With rain and heat during the same period, strong erosion commonly happened in Asian monsoon region when vegetation flourished in the wet season, which likely resulted that more organic carbon were washed into rivers^10,11^. Organic carbon ratios of suspended solids in the 11 rivers were investigated in China^12^, which indicated that the riverine organic carbon ratios in southern China were observably high. Most researches on riverine organic carbon were carried out in Yangze River^13–15^, Yellow River^16^ and Xijiang River^10,11,17^, *et al*. However, there were very few reports about the output processes of riverine organic carbon in the tropical region of China. In this paper, riverine organic carbon was investigated in Changhuajiang River (CHJR) basin, locating at the mid-west of Hainan Island, China with the aim of understanding the materials fluxes originating from Hainan Island into the South China Sea.

## Study Area

**Figure 1:**
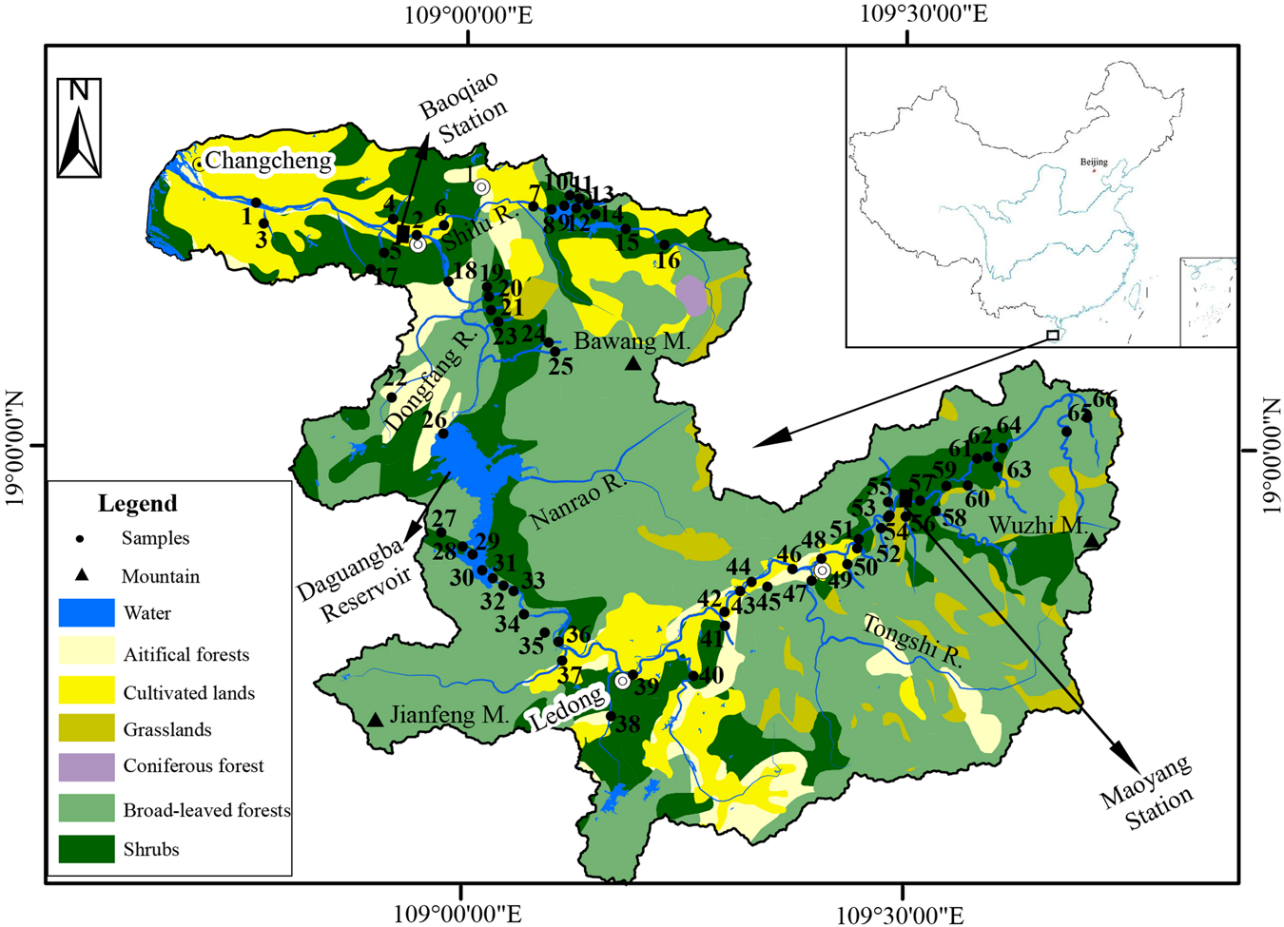
The sketch of vegetation types and distribution of samples in the CHJR basin (Made with ArcGIS 10.0).

Originating from the central of Hainan Island, the CHJR initially flowed to southwest and then turned to northwest in the Ledong County. It finally drained into the South China Sea at Changcheng County (Fig. 1). The CHJR was the second largest river in Hainan Island, with the 5150 km^2^ area and 232 km length. This basin was located in the Asian tropical monsoon, where the climate was obviously divided into wet seasons (from May to October) and dry seasons (from November to April of the second year). The annual temperature in the whole basin ranged from 17.70 °C to 27.93 °C. The average rain of multi-year was up to 1508.55 mm, with 83.55% in the wet season. The CHJR discharges 22.3 × 10^8^ m^3^ water (at the Baoqiao hydrologic station, representing 90% of the total basin area), accounting for 53.48% of the average runoff (41.7 × 10^8^ m^3^) over years^18^. Their 18.7 × 10^8^ m^3^ was exported in wet season and 3.6 × 10^8^ m^3^ in dry season.

The Indosinian and Variscan granites including biotite-monzonite granite, granodiorite, quartz-diorite and granite-porphyry were widely exposed, which represent the 66.84% of total area. Tropical monsoon forests were predominant in the middle and upper basin, and the main crops (banana and cane, etc.) and artificial economic forests (eucalyptus, rubber and mango, etc.) were largely distributed in the middle and lower reach. From the basin boundary to main valley, the soil types showed a gradual transition of yellow soil-red soil-red cinnamon soil, accounting for 18.69%, 27.71% and 53.62% of the total drainage area, respectively. The soil organic carbon contents were high in Bawang Mountain and Jianfeng Mountain. The mountain and hilly, with the steep terrain, were mainly distributed in middle and upper reaches. In addition, the platform and plain were mainly distributed in main valley and downstream alluvial areas.

## Sampling and Experiment

### Sampling

66 water samples (A) within the CHJR basin were collected in January (dry season) and August (wet season) 2014 from the river mouth to the headwater and were positioned by the GPS (Fig. 1). About 25 L water samples (B) were collected at Baoqiao and Maoyang hydrologic stations, representing for lower and upper reaches, respectively. Temperature, pH, conductivity (Cond) and total dissolved solid (TDS) were monitored by Ultrameter™ Model 9 P with the accuracy of ±0.10 °C, ±0.20, ±1 µS/cm, and ±0.2 mg/L *in situ*.

### Experiments and methods

After being filtered through 0.45 μm-porosity Whatman GF/F filters, the water samples A were measured by TOC-VCPH analyzer, Shimadzu Co., Japan, referring to water quality-determination of TOC by nondispersive infrared absorption method (GB13193-91). Measured accuracy maintained in the range of −0.01 mg/L~ 0.01 mg/L. All samples were kept in the refrigerator at 4 °C before analysis. The water samples B were pump filtered through 150 mm diameter cellulose acetate membranes with 0.45 μm-porosity. Particulate matters residual in those membranes were gathered in evaporating dishes and dried at 4 °C about 2~3 days. They then were weighed for TSS with the balance (0.1 mg~1 g). And the contents of organic carbon (POC) and nitrogen (PON) were measured by the element analyzer ELCHNS-O, VariO Co., German, with the precision of 0.3%.

The soil organic carbon contents in the basin were calculated by the weight average of different vegetation types, ranging from 0 to 12.61 kg/m^3^. Soil organic carbon contents in tropical rain forest, artificial economic forest and agricultural cultivation was the measured by our research group^19^, and those in tropical shrub and grassland were cited from the literature^20–22^. The slope information was converted according to the DEM data (http://gre.geodata.cn/) in the basin.

## Results

### DOC concentrations and spatial-temporal variation in CHJR

**Figure 2:**
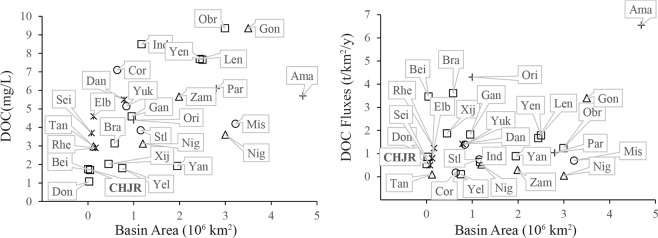
The DOC concentration, DOC Fluxes and basin area of the major rivers in global. (Yan = Yangtze River^13^; Yel = Yellow River^3^; Bra = Brahmaputra^3^; Gan = Ganges River^3^; Ind = Indus River^3^; Ori = Orinoco River^3^; Par = Paraná River^3^; Zam = Zambezi river^3^; Nil = Nile River^3^; Dan = Danube River^3^; Sei = Seine^3^; Xij = Xijiang River^10^; Don = Dongjiang River^23^; Bei = Beijiang River^24^; CHJR, This study; Len = Lena River^25^; Obr = Ob River^26^; Yen = Yenisei River^26^; Cor = Colorado River^27^; Mis = Mississippi River^28^; Stl = St Lawrence River^29^;Yuk = Yukon River^30^; Ama = Amazon River^31^; Con = Congo River^31^; Nig = Niger River^31^; Tan = Tana River^32^; Elb = Elbe^33^; Rhe = Rhein^33^).

DOC concentrations in the CHJR ranged from 0.22 mg/L to 11.75 mg/L with an average of 1.75 ± 1.91 mg/L, which was lower than the average value (5.75 mg/L) of global rivers^1^ (Fig. 2). Comparing with other rivers in the Asia, the DOC concentrations were far lower than those rivers flowing into Arctic Ocean in the north of Asia and gentle lower than the main large rivers in China, such as Yangtze River, Yellow River and Xijiang River, etc. Not only that, the DOC concentrations were also lower than those rivers located in tropical regions. CHJR basin was provided with high temperatures and rapid decomposition rates of organic matters. Meanwhile, low intensity of human activities maybe was another reason for the low DOC concentrations in the CHJR.

**Figure 3:**
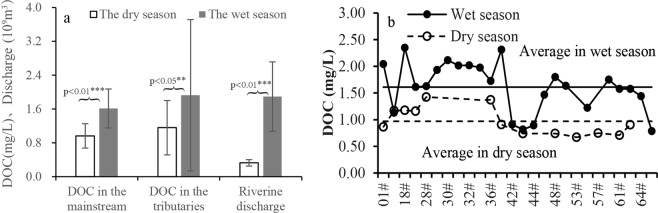
The seasonal difference of riverine DOC concentration and discharge and those spatial variation in the mainstream.

The riverine DOC concentrations had significant difference between the dry season and wet season, which were consistent with seasonal changes of riverine discharge (Fig. 3a). Moreover, the average riverine DOC concentrations in the wet season were higher than those in the dry season. This phenomenon both appeared in mainstream and tributaries. Riverine DOC was commonly divided into the exogenous and endogenous sources. The former primarily came from terrestrial materials (plants and soils) erosion, and the latter was produced during the growth of aquatic phytoplankton^1^. Due to the weather that hot and rainy in the wet season, more exogenous DOC was transported into rivers under intense erosion. At the same time, the vigorous growth of aquatic phytoplankton contributed more DOC endogenous sources.

No matter in the dry season or the wet season, the average of riverine DOC concentrations in the tributaries was higher than that in the mainstream. Stemming from mountains with steep terrain, the tributaries had taken in more material from terrestrial ecosystem. Organic matters were decomposed when they were transferred to the mainstream, with slow-flowing water conducive to microbial activity. The dilution effect may be another reason for the low riverine DOC concentration in mainstream.

The riverine DOC concentrations were higher in the middle reaches (30#~39#) than those in the upper and lower reaches (Fig. 3b), and that phenomena both happened in the dry season and the wet season. The construction of DGB reservoir in the basin had changed the hydrological situation and largely multiplied the aquatic algae^34–36^, which increased the endogenous sources of DOC in middle reaches. After discharging from DGB reservoir, the riverine DOC concentration gradually decrease towards to the lower reaches (30#~26#), which was likely because that the impact of reservoir gradually weakened.

### The POC concentrations and spatial-temporal variation in CHJR

**Figure 4:**
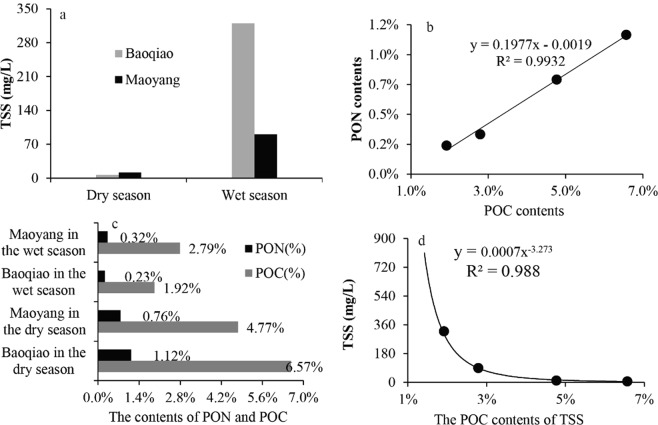
The contents and correlations of TSS, PON, POC in CHJR.

TSS in rivers was the main carrier of POC. Therefore the POC exporting pattern was affected by the TSS erosion process to some extent. Terrestrial ecosystem suffered from strong erosion with heavy rain in wet season, and terrigenous materials were largely transported into the river. Above analysis likely was the main reason that caused the riverine TSS contents were far higher in the wet season than those in the dry season (Fig. 4a). The TSS content in the upstream (Maoyang Station) was higher than that in the downstream (Baoqiao Station) in the dry season, however, which was contrary in the wet season. Due to the impact of seasonal water storage in the DGB Reservoir, the flow rates significantly decreased after injection into the reservoir in the dry season, which brought that a considerable part of TSS was detained in reservoir. However, the reservoir regularly discharged and maintained at a certain capacity in order to prevent floods in the wet season.

Beside of POC, PON was also important ingredient of riverine suspended solid. POC and PON contents in the CHJR water simultaneously changed with a linear trend (Fig. 4b), which illustrated that they had the same source. There was a largely seasonal variation in POC contents, which in the dry season was 2.41 times of wet season (Fig. 4c) and obviously increased with the reduction of TSS contents (Fig. 4d). For that phenomenon, the possible reasons were speculated as follows. The surface soil was the important source of riverine suspended matter. The soil POC of main vegetation types in the CHJR basin had significant seasonal difference, which in the dry season (0.82%) was 1.71 times of those in the wet season (0.48%)^37^. Meanwhile, the POC content in soil profile gradually decreased from top to bottom, so the POC content of suspended matter in the river decreased with the erosion strengthening in the wet season. Furthermore, the increase of TSS reduced transmittance in the river, which largely inhibited the photosynthesis in aquatic ecosystem and also brought the reduction of endogenous POC.

## Discussion

### Riverine dissolved organic carbon hydrodynamic output process

The Baoqiao hydrologic station, representing 90% of the total basin area, was selected for the control section of the whole basin, where the DOC concentrations were respectively 1.17 mg/L and 1.13 mg/L in the dry season and wet season. According to Eq. (1), the total output amount of riverine DOC was calculated as 2.53 × 10^3^ t/y with 83.40% in the wet season and 16.60% in the dry season, and the above-mentioned flux was converted to 0.55 t/km^2^/y in consideration of the basin area (Eq. (2)). The DOC flux in CHJR was lower than that in large rivers such as the Amazon and Congo rivers etc. (Fig. 2) and the average DOC flux 2.04 t/km^2^/y in most global rivers^4^. Due to influence of damming in the basin, the CHJR runoff (22.2 × 10^8^ m^3^) in the recent years only accounted for 53.24% of the multi-years runoff (41.7 × 10^8^ m^3^). If the multi-years runoff was taken into account, the average DOC flux in the CHJR was up to 1.03 t/km^2^/y, which was higher than those of Yangtze River and Dongjiang River in China and most rivers in the North America and Africa (Fig. 2). Consequently, the dams could reduce the carbon transport capacity of rivers at some extent.

1 $${{F}}_{{DOC}}=\mathop{\sum }\limits_{{i}=1}^{2}{{\rm{[}}\text{DOC}{\rm{]}}}_{{i}}\times {{Q}}_{{i}}$$

2 $${{f}}_{{\rm{DOC}}}=[{F}_{{\rm{DOC}}}]/S$$

(Where *F*_*DOC*_ was the total DOC output amount and *f*_DOC_ represented the DOC fluxes. *Q* and S denoted the discharges and the area of the CHJR basin, respectively. The *i* = 1, 2 meant the dry and wet season).

### Influencing factors of organic carbon output in CHJR

According to the previous scholars, soil organic carbon, vegetation coverage, runoff depth and slope information were key factors controlling the output fluxes of riverine organic carbon^4^. Seven tributaries in CHJR basin were selected for studying the factors influencing riverine organic carbon. Subject to sampling and analysis conditions, the riverine DOC in the CHJR were mainly discussed here. The DOC concentrations of seven tributaries were the weighted average values according to runoff in the dry and wet season. The DOC fluxes of seven tributaries were calculated by Eqs (1) and (2).

**Figure 5:**
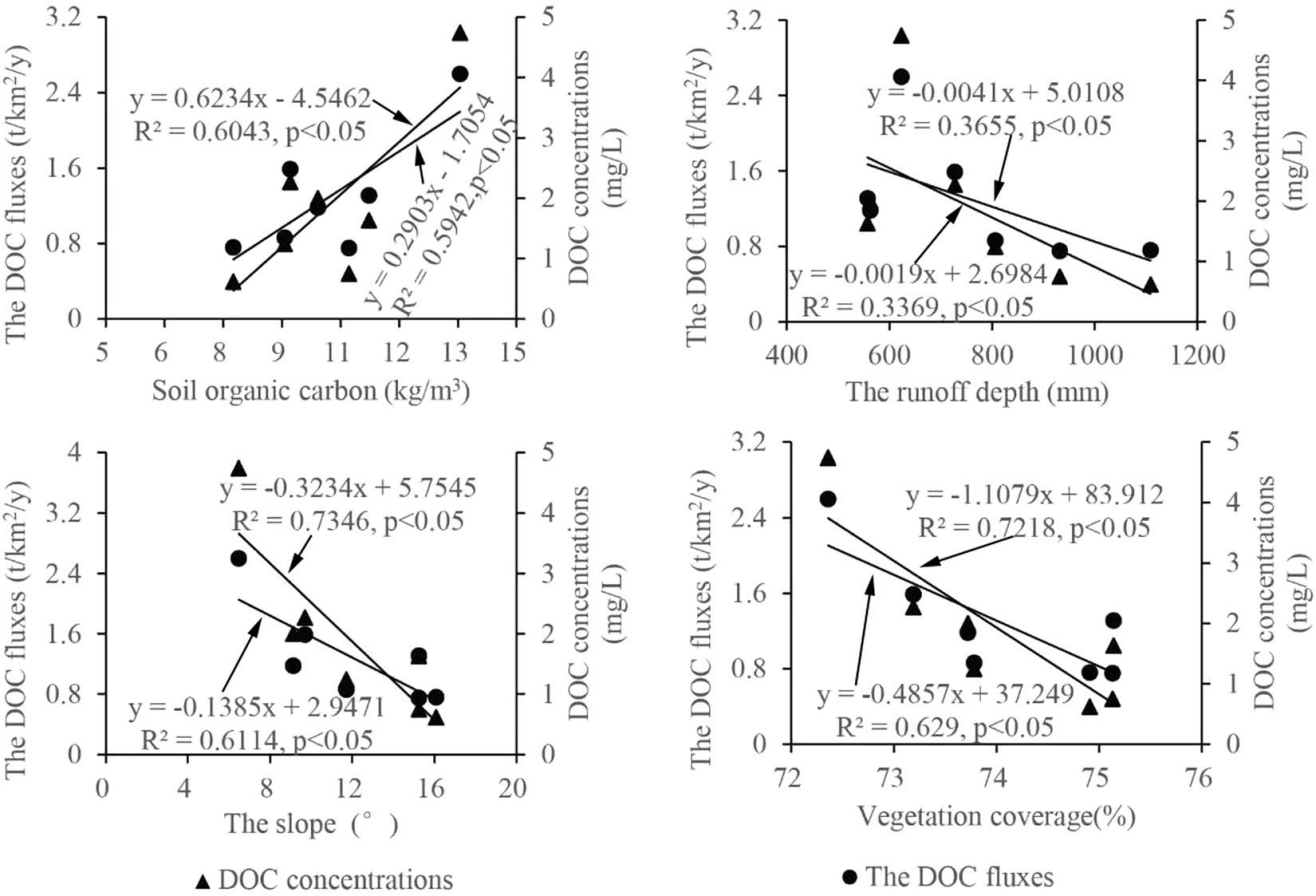
Influencing factors of organic carbon output in CHJR.

The relationships among DOC concentrations, DOC fluxes and influencing factors including soil organic carbon, runoff depth, slope and vegetation coverage were analyzed (Fig. 5). Results displayed that significantly positive relationships (p < 0.05) between DOC concentrations (DOC fluxes) and soil organic carbon contents were found (Fig. 5a) in the CHJR basin. However, the opposite case that significantly negative relationships (p < 0.05) existed between DOC concentrations (DOC fluxes) and the other three factors including runoff depth, slope and vegetation coverage (Fig. 5b–d). As many scholars had confirmed that soil organic carbon was the primary source of riverine organic carbon^1,10,25^, riverine DOC concentrations and fluxes commonly increased with the increase of soil organic carbon contents. Numerous studies had revealed that slope was the key factor affecting the runoff speed^38,39^. Water retention time in terrestrial ecosystem was short in steep mountainous areas with high slope due to the fast water convergence rate. The interaction between water and soil in the basin was not sufficient, which brought that few soil carbon was carried into rivers. The statistics showed that runoff depth negatively correlated with soil organic carbon and positively correlated with slope in the tributaries. Therefore, the relationship between DOC concentrations (DOC fluxes) and runoff depth was largely influenced by soil organic carbon and slope in the basin. The tributaries with the higher runoff depth tended to be provided with the higher soil organic carbon contents and lower slope according to above analysis. The existence of surface vegetation could effectively reduce soil erosion by the interception of stems and leaves on rainfall and the consolidation effect of roots on soil^35^, which largely reduced the terrestrial sources of riverine organic carbon.

**Figure 6:**
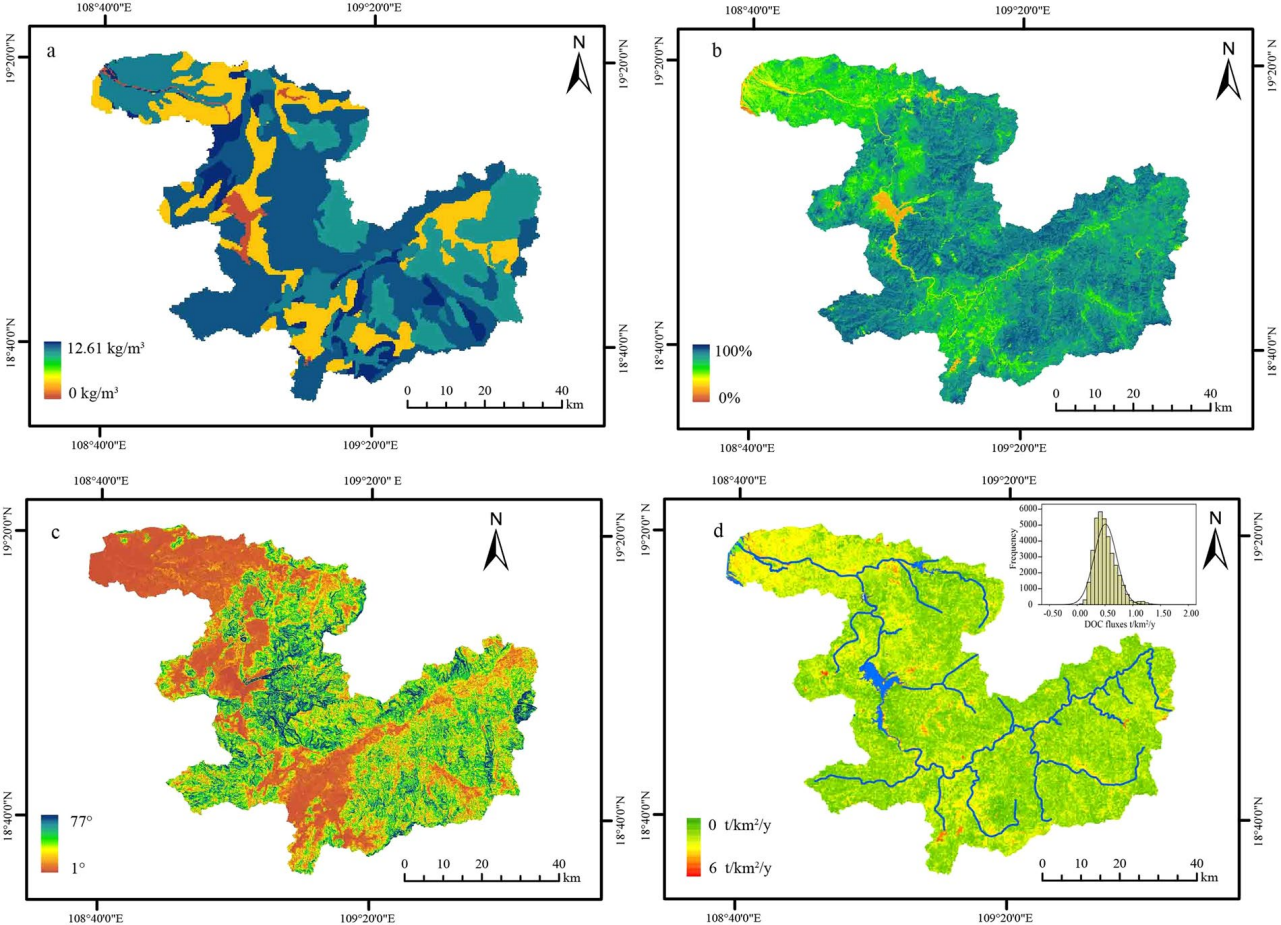
The soil organic carbon (**a**), slope (**b**), vegetation coverage (**c**) and simulated DOC fluxes (**d**) in CHJR basin (Analyzed by SPSS18.0).

Based on the relationships between DOC fluxes and its influencing factors (including the soil organic carbon, slope and vegetation coverage), a linear model was established to calculate the DOC fluxes by the SPSS18 (Eq. (3)). The vegetation coverage in the basin positively correlated with the slope largely due to the human activities (Fig. 6a–c). According to the linear model and above environment information in CHJR basin, the grid units of DOC fluxes were simulated (Fig. 6d). The result displayed that the DOC fluxes in the basin ranged from 0 t/km^2^/y to 6 t/km^2^/y and its frequencies were normally distributed with the central axis of 0.52 t/km^2^/y. Meanwhile, the weighted average of DOC fluxes was calculated as 0.49 t/km^2^/y, gently lower than the measured value 0.55 t/km^2^/y.

Ludwig *et al*. had established the watershed DOC fluxes model in consideration of runoff depth, slope and soil organic carbon contents^4^ *(*Eq. (4)). The average runoff depth was 479.97 mm based on the recent five years runoff in the CHJR basin. The average organic carbon content was 10.05 kg/m^3^ according to the measured values by our research group^19^. The average slope 0.27 radians (15.33°) were statistically obtained by ArcGis 10.0 in CHJR basin. The DOC flux was calculated as 0.51 t/km^2^/y based on the Eq. (4) close to the value of 0.49 t/km^2^/y, which illustrated that the Eq. (4) was reliable in CHJR basin.

3 $${{f}}_{{\rm{DOC}}}=0.61\times {C}_{soil}+0.624\times S-1.148\times V,\,{r}=0.89,\,{p} > 0.05$$

(This study)

4 $${f}_{DOC}=0.004Q-8.76S+0.095{C}_{soil},\,{r}=0.\,90,\,{p} > 0.\,01$$

(Where *f*_DOC_ was the DOC flux (t/km^2^/a). Q, S, C_soil_ and V denoted the runoff depth (mm), slope (radians), soil organic carbon contents (kg/m^3^) and vegetation coverage, respectively^3^).

### Riverine particulate organic carbon hydrodynamic output process

According to the measured values at the Baoqiao and Maoyang hydrological stations, the average TSS was calculated as 107.06 mg/L in the CHJR, which was basically consistent with the monthly average from 1957 to 2008^40^. By the Eq. (5), the TSS output amount in CHJR basin was 38.72 × 10^4^ t in 2014, which was much lower than the multi-years average 69.91 × 10^4^ t^40^. Considering that the average runoff in the recent five years accounted for 53.24% of the multi-years, the above analysis was credible. Accordingly, the TSS output flux was 41.08 t/km^2^, and the surface erosion rate was calculated as 82 m/Ma in light of the average soil density 1.83 g/cm^3^ in the basin (measured by our research group^19^), which illustrated that CHJR basin had long been in mechanical denudation.

5 $${{F}}_{{TSS}}=\mathop{\sum }\limits_{i=1}^{2}{[TSS]}_{i}\times {Q}_{i}$$

(Where *F*_*TSS*_ was the total TSS output amount and Q represented the discharges of the CHJR basin. The *i* = 1, 2 meant the dry and wet season).

**Figure 7:**
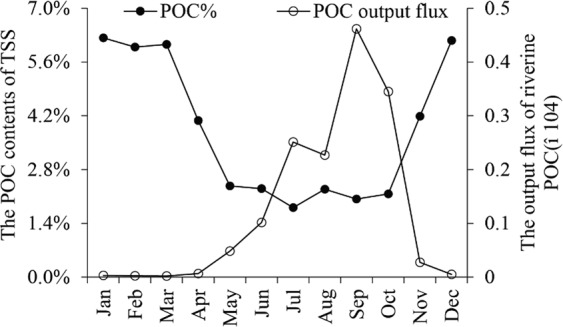
The monthly POC contents and output amount in multi-years.

Despite that the POC contents (POC%) of TSS were relatively low in the wet season, but the POC concentrations (*C*_*POC*_) were high, because the TSS concentration in the wet season was 22.51 times of that in the dry season (Fig. 4a). According to Eqs (6) and (7), about 8.28 × 10^3^ t POC were annually exported by CHJR, 98% of which happened in the wet season. Based on the exponential function between TSS and POC (Fig. 4d), the contents and output fluxes of POC were simulated (Fig. 7). The simulation results displayed that POC% kept at relatively low level from May to October and their monthly average was calculated as 3.81%, approximating to our measured value 4.02%. The variation of monthly POC output fluxes was consistent with the CHJR’s runoff and opposite to the POC%. Riverine POC was largely exported from June to October in CHJR, accounting for 93.60% of the total amount. This phenomenon confirmed the previous conclusions from our research group that riverine POC in the Asian monsoon regions was mainly exported during the summer^10^.

6 $${C}_{POC}=TSS\times POC \% $$

7 $${F}_{POC}=\mathop{\sum }\limits_{i=1}^{2}{[{C}_{POC}]}_{i}\times {Q}_{i}$$

(Where FPOC was the total POC output amount and fPOC represented the POC fluxes. CPOC and TSS were the concentration of POC and TSS in the river (mg/L) and POC% represented the contents of POC in the TSS (%). The meanings of Q, S and i were described in the previous section).

### The erosion environment of terrestrial organic carbon

The riverine POC flux in CHJR basin was calculated as 1.78 t/km^2^/y (Eq. (7)), which was gently higher than the average of global rivers (1.65 t/km^2^/y)^3^ and far lower than those in other large rivers in domestic, such as 6.14 t/km^2^/y in Yangtze River^4^, 14.68 t/km^2^/y in Yellow River^41^ and 8.30 t/km^2^/y in Xijiang River^10^. The previous studies have shown that there were high soil erosion modulus those basins^42^. Riverine POC fluxes were related to the surface soil erosion conditions. When the surface environment of the basin was dominated by leaching, water stayed for a long time in the surface soil. After long-term filtration, the large-size particle matters were likely intercepted in the terrestrial ecosystem, which was beneficial to the production of riverine DOC. Otherwise, when the surface environment of the basin was dominated by scouring, more soil organic carbon were transported into rivers with the particulate matters^10^. Accordingly, the ratios of POC fluxes and DOC fluxes were the important indexes reflecting the surface environment in the basin. On the basis of the POC fluxes (1.78 t/km^2^/y) and DOC fluxes (0.55 t/km^2^/y) in CHJR, the ratio was up to 3.24, which was far lower than that (33.33) in the Yellow River^41^ and relatively close to 4.35 in the Xijiang River^10^. However, it was far higher than the average (0.81) of global rivers^4^. In that sense, the surface environment was dominated by scouring in CHJR basin.

### The source of riverine organic carbon in CHJR

**Figure 8:**
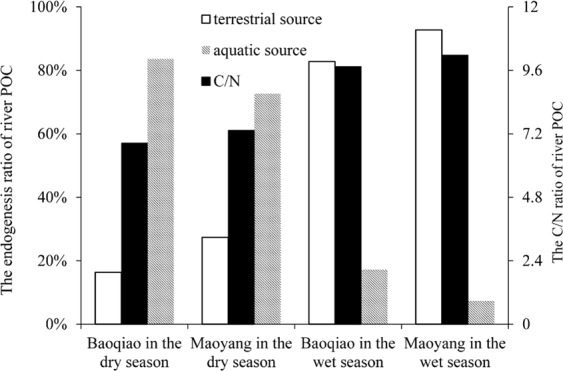
The source and C/N ratios of riverine organic matter.

Riverine organic matters were usually the mixture of terrestrial and aquatic ecosystems. In previous studies, C/N ratios of organic matter were widely used as the important indexes to trace the riverine organic carbon source^43–45^. There were high C/N ratios of organic matter in the wet season in CHJR, comparing with those in the dry season (Fig. 8). And the C/N ratios in the upper reach were always higher than those in the lower reach both in wet season and dry season. The formula about the source of riverine organic carbon was established, taking the C/N ratios of organic matter in terrestrial and aquatic ecosystems as two end members (Eq. (8)). Based on different types of land use, the average C/N ratio in terrestrial ecosystems was calculated as 10.49 in CHJR basin (Unpublished). The C/N ratios of organic matter in aquatic ecosystems was referred to that of Zengjiang Basin in South China^46^, taking the similar environment of two basins into account. Accordingly, the source of riverine organic carbon in CHJR was analyzed by the Eq. (8).

8 $${({C}/{N})}_{{sample}}={f}\times {({C}/{N})}_{{land}}+(1-{f})\times {({C}/{N})}_{{aquatic}}$$

(Where the *f* was the ratios of terrestrial source).

Analysis results showed that there were 82.78% of riverine organic matter from terrestrial ecosystem in Baoqiao Station in the wet season. In Maoyang Station, the ratio of riverine organic matter from terrestrial ecosystem was up to 92.70% in the wet season. It was deduced that terrestrial ecosystem was the main source of riverine organic matter in the wet season in CHJR basin. Correspondingly, about 83.63% of riverine organic matter was produced from aquatic ecosystem in the Baoqiao Station in the dry season. And aquatic ecosystem contributed 72.65% of riverine organic matter in the Maoyang Station in the dry season. From this, it was illustrated that aquatic ecosystem was the main source of riverine organic matter in the dry season in CHJR basin. As mentioned above, terrestrial particles were largely transported into the river in the wet season, due to abundant rainfall and strong soil erosion. At the same time, the increase of particle suspended solids would reduce the transparency of water bodies, which inhibited the photosynthesis of aquatic ecosystem. On the contrary, the surface erosion was weakened, and the photosynthesis of aquatic ecosystem strengthened in the dry season. There were high ratios of terrestrial source in the upper reach no matter in the wet season or the dry season. Relatively speaking, the surface erosion was strong in the upper reach with fast flow rate in mountainous areas, which likely brought more terrestrial materials.

Based on the above ratios of riverine organic matter, POC output amount from terrestrial ecosystem and aquatic ecosystem was calculated by Eqs (9 and 10). Among 8.28 × 10^3^ t POC exported in CHJR basin, about 7.15 × 10^3^ t originated from terrestrial ecosystem and 1.13 × 10^3^ t stemmed from aquatic ecosystem. About 87.74% terrestrial organic carbon was exported in the wet season, and 12.26% in the dry season. Conversely, about 78.14% aquatic organic carbon was produced in the dry season, and 21.86% in the wet season.

9 $${[{F}_{POC}]}_{land}=\mathop{\sum }\limits_{i=1}^{2}{[{F}_{POC}]}_{i}\times {Q}_{i}\times f$$

10 $${[{F}_{POC}]}_{aquatic}=\mathop{\sum }\limits_{i=1}^{2}{[{F}_{POC}]}_{i}\times {Q}_{i}\times (1-f)$$

### The significance of riverine organic carbon exporting in global

**Figure 9:**
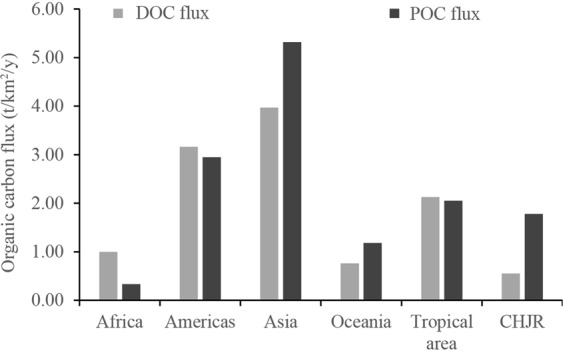
The organic carbon fluxes comparison between CHJR and other tropical rivers^7^.

According to the above analysis, CHJR delivered approximately 10.81 t organic carbon to the estuaries annually, of which, 30.56% was DOC and 69.44% was POC. The DOC flux of CHJR was lower than those of tropical (30°N–30°S) rivers in Africa, America, Asia and Oceania (Fig. 9) given by Huang *et al*.^7^. While, the POC flux was lower than those in America and Asia and close to the flux average of tropical rivers. Comparing with other tropical rivers, there was higher ratio of POC flux to DOC flux in CHJR, which illustrated that the hydrodynamic output process of riverine organic carbon represented a special surface erosion process in Asian tropical monsoon. The organic carbon fluxes were likely overestimated, taking the wide area of tropical monsoon in Asia. It was certified that the organic carbon removal phenomena commonly occurred in the estuaries^47,48^. Based on existing research, Dai *et al*. adopted an average removal rate of 10% for estuarine organic carbon^6^. Considering the above results, we obtained an estimate of DOC flux of 9.73 t/y in CHJR basin, however, which should be furtherly studied due to lack of relevant material.

## Conclusion

Transport fluxes and properties of riverine organic carbon in CHJR located at tropical monsoon region were discussed. DOC concentrations ranged from 0.22 mg/L to 11.75 mg/L with an average of 1.75 mg/L in the CHJR, which was lower than those in most global rivers. There were high riverine DOC concentrations in the wet season and large variation of riverine DOC concentrations in the mainstream. Due to the effect of damming in the basin, riverine DOC concentrations were higher in the middle reach than those in the upper and lower reach. Output flux of riverine DOC was calculated as 0.55 t/km^2^/y, which could be revised up to 1.03 t/km^2^/y, considering that the riverine discharge before dam construction. Soil organic carbon, runoff depth, slope and vegetation coverage were the key factors controlling output fluxes of riverine organic carbon, and a linear model suitable in CHJR basin was established in this paper. The POC contents of TSS had a largely seasonal variation in the CHJR, which in the dry season were 2.41 times higher than that in the wet season. From the perspective of spatial variation, POC contents of TSS in the upper reach were higher than those in the lower reach in the wet season, and this spatial distribution pattern was the opposite in the dry season, due to the impact of seasonal water storage in the DGB Reservoir. About 8.28 × 10^3^ t POC were annually exported by CHJR, and riverine POC flux in the basin was calculated as 1.78 t/km^2^/y. The POC flux was higher than the average of global rivers but far lower than those in other domestic larger rivers. About 7.15 × 10^3^ t POC was annually scoured into the river from terrestrial ecosystem, and 1.13 × 10^3^ t POC stemmed from aquatic ecosystem. Meanwhile, 87.74% of terrestrial source POC were exported in the CHJR basin during the wet season. This research confirmed that the surface erosion of terrestrial ecosystem in the wet season was main contributor to riverine organic carbon in the CHJR basin.
